# Running Speed in Mammals Increases with Muscle n-6 Polyunsaturated Fatty Acid Content

**DOI:** 10.1371/journal.pone.0000065

**Published:** 2006-12-20

**Authors:** Thomas Ruf, Teresa Valencak, Frieda Tataruch, Walter Arnold

**Affiliations:** Research Institute of Wildlife Ecology, University of Veterinary Medicine, Vienna, Austria; University of Stellenbosch, South Africa

## Abstract

Polyunsaturated fatty acids (PUFAs) are important dietary components that mammals cannot synthesize *de novo*. Beneficial effects of PUFAs, in particular of the n-3 class, for certain aspects of animal and human health (e.g., cardiovascular function) are well known. Several observations suggest, however, that PUFAs may also affect the performance of skeletal muscles in vertebrates. For instance, it has been shown that experimentally n-6 PUFA-enriched diets increase the maximum swimming speed in salmon. Also, we recently found that the proportion of PUFAs in the muscle phospholipids of an extremely fast runner, the brown hare (*Lepus europaeus*), are very high compared to other mammals. Therefore, we predicted that locomotor performance, namely running speed, should be associated with differences in muscle fatty acid profiles. To test this hypothesis, we determined phospholipid fatty acid profiles in skeletal muscles of 36 mammalian species ranging from shrews to elephants. We found that there is indeed a general positive, surprisingly strong relation between the n-6 PUFAs content in muscle phospholipids and maximum running speed of mammals. This finding suggests that muscle fatty acid composition directly affects a highly fitness-relevant trait, which may be decisive for the ability of animals to escape from predators or catch prey.

## Introduction

Comparative studies on locomotor performance and maximum running speed (MRS) in mammals have mostly concentrated on biomechanical models. Factors such as skeletal structure, gravity, bone-length and width, or peak stress on muscles and tendons affect MRS and explain several differences in locomotor performance of mammals of different body size [1], [2]. Research on muscles specialized on high-frequency contractions, such as the tail shaker muscle of rattlesnakes, has revealed that on the cellular level, muscle cell performance can be modified by both protein isoforms (of troponin, myosin, and ion pumps) and quantitative adaptations of the densities of sarcoplasmatic reticulum (SR) and mitochondrial membranes [3]. To our knowledge, however, it has not been investigated previously whether these membranes also may show systematic qualitative changes concerning their fatty acid composition, namely PUFA content or relative abundance of the n-3 and n-6 PUFA subclasses (which have their first double bond at the 3^rd^ and 6^th^ carbon of the fatty acid chain, respectively), that could be associated with MRS across mammals. Several previous studies suggest that PUFAs may indeed affect the performance of skeletal muscles and locomotion in vertebrates. In Atlantic salmon, for instance, dietary fatty acid composition and resulting changes in muscle lipid composition significantly affected maximum swimming speed. These effects were largely due to a positive relation between swimming speed and the most common n-6 fatty acid in muscle lipids, linoleic acid (C18:2n-6) [4]. Also, muscles that undergo very high contraction frequencies, such as the pectoral muscle in hummingbirds, contain very large amounts of docosahexanoic acid (DHA, C22:6n-3), a highly unsaturated PUFA [5]. In humans, training exercise increases muscle PUFA content [6]. Further, we recently found that the proportions of PUFAs in the muscle phospholipids of an extremely fast runner, the brown hare (*Lepus europaeus*), are very high compared to other mammals [7].Therefore, we hypothesized that muscle phospholipid profiles may be functionally related to locomotor performance, and were interested to see if there is a relation between either total PUFA content, any of the PUFA subclasses, or a particular fatty acid, and locomotor function as measured by running speed.

## Results

**Figure 1:**
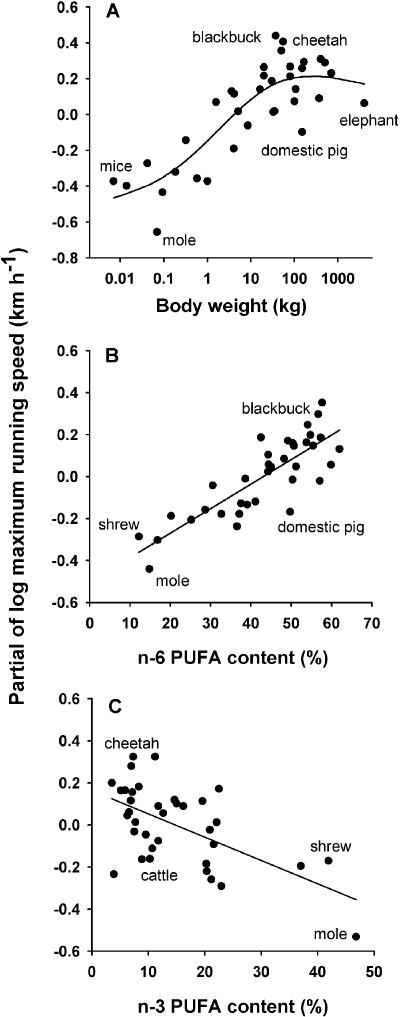
Maximum running speed in mammals. (A) MRS as a function of body weight. (B) The relation of MRS to n-6 PUFA content, and to (C) n-3 PUFA content in phospholipids of skeletal muscles. Shown are partial residual plots (i.e. the independent effect of each predictor variable) from general additive mixed models using a smooth term for body weight (estimated df = 2.66, F = 4.77, P = <0.001) and linear terms for n-6 and n-3 PUFA content. To adjust for phylogenetically caused correlations models included the family membership of each species as a random factor.

MRS increased with body weight in a nonlinear manner (Fig. 1A) and levelled off among very large mammals. After adjusting for this body size effect we found that MRS increased significantly with the proportion of total muscle phospholipid PUFAs (Table 1). This association was, however, almost entirely due to a positive relation between MRS and the n-6 PUFA class only, which was the best predictor of MRS in this comparison (Fig. 1B, Table 1). Accordingly, among individual fatty acids, contents of the two most common fatty acids of the n-6 class, Linoleic acid (C18:2n-6) and Arachidonic acid (C20:4n-6) were also positively related to MRS (Linoleic acid: slope = +0.015, t = +3.113, P = 0.0038; Arachidonic acid : slope = +0.012, t = +2.10, P = 0.043). Species with specialized morphological adaptations designed for extremely high running speeds, such as the cheetah (*Acinonyx jubatus*) and blackbuck (*Antilope cervicapra*), are still much faster than predicted from body size and phospholipid n-6 PUFA content alone (Fig. 1), but overall, the relation between muscle membrane composition and MRS was surprisingly strong. When back-transformed to actual velocities, the regression line in Fig. 1B indicates that predicted MRS increased from 13.0 to 49.2 km h^−1^ as n-6 PUFA content increased from 12.2 to 61.5%. This corresponds to a speed gain of 0.734 km h^−1^ for each percent increase in the proportion of n-6 PUFAs.

**Table 1:**
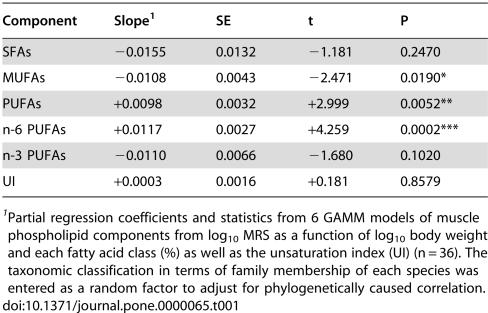
Regression results for the relation between phospholipid fatty acid components and MRS.

Component	Slope^1^	SE	t	P
SFAs	−0.0155	0.0132	−1.181	0.2470
MUFAs	−0.0108	0.0043	−2.471	0.0190*
PUFAs	+0.0098	0.0032	+2.999	0.0052**
n-6 PUFAs	+0.0117	0.0027	+4.259	0.0002***
n-3 PUFAs	−0.0110	0.0066	−1.680	0.1020
UI	+0.0003	0.0016	+0.181	0.8579

1 Partial regression coefficients and statistics from 6 GAMM models of muscle phospholipid components from log_10_ MRS as a function of log_10_ body weight and each fatty acid class (%) as well as the unsaturation index (UI) (n = 36). The taxonomic classification in terms of family membership of each species was entered as a random factor to adjust for phylogenetically caused correlation.

The relation between MRS and the proportion of n-3 PUFAs, on the other hand, was slightly negative (Fig. 1C), but due to a large scatter in n-3 PUFA abundance, this association was statistically not significant (Table 1). Because n-3 PUFA content dominated species-specific differences in the degree of fatty acid unsaturation, the unsaturation index (UI, i.e. the total number of double bonds per 100 acyl chains), was also unrelated to MRS (Table 1). However, since low levels of n-6 PUFAS were largely replaced by n-3 PUFAs and vice versa (r = −0.71, P<0.001) it is not surprising that the single best predictor of MRS was the difference between n-6 PUFA and n-3 PUFA content (slope+0.0102, t = +4.559, P<0.0001).

**Figure 2:**
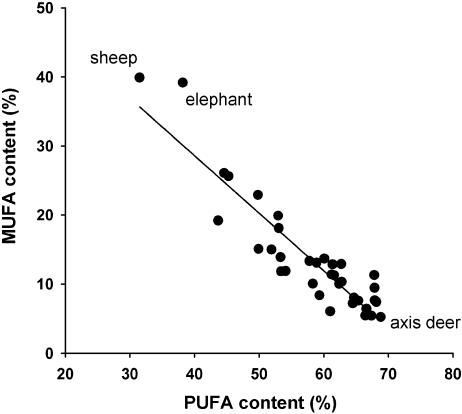
Phospholipid MUFAs increase as PUFAs decrease. Skeletal muscle MUFA content was highly determined by PUFA content (% MUFA = 64.18−0.872×% PUFA; t = −14.76, P<0.0001; R2 = 0.865, n = 36.)

MRS was not significantly affected by saturated fatty acid (SFA) content, but decreased as the proportion of monounsaturated fatty acids (MUFAs) increased (Table 1). This negative relation between MRS and MUFAs was not independent of the impact of PUFAs however, because MUFA content was negatively correlated with the abundance of both n-6 PUFAs alone (r = −0.549, P<0.001) and, even more strongly, with total PUFA content (r = −0.930, P<0.0001, Fig. 2). The saturated fatty acid (SFA) content, on the other hand, varied much less than the other membrane constituents and showed a considerably weaker correlation with PUFA content (r = −0.352, P = 0.035).

We found no evidence for the possibility that our finding of a positive relation between MRS and n-6 PUFAs, or the inverse relation to MUFAs, could have been caused by the inclusion of species with a low number of specimens in which fatty acid profiles were determined. A bootstrap analysis based on random combinations of single specimens clearly confirmed the statistical significance of associations between n-6 PUFAs, total PUFAs, and MUFAs (Protocol S2).

The above results on the relation between muscle phospholipid fatty acid composition and MRS were further corroborated by phylogenetic GLS regression models (see Material and Methods, using linear terms for both body weight and phospholipid components). Partial regression coefficients from phylogenetic models for the effects of PUFAs, n-6 PUFAs, and MUFAs were statistically significant (Protocol S3) and showed the same direction of effects and similar effect sizes as indicated by our GAMM approach of data analysis. Phylogenetic GLS models indicated, however, that due to a high degree of similarity within clades, body weight had no independent effect on MRS when adjusted for phylogenetic distances between species. Partial regression coefficients for log_10_ body weight from 6 models (testing for the effects of different fatty acids and UI) ranged from −0.005 to +0.049 and were not significantly different from zero (0.39<P<0.91).

## Discussion

Our data provide strong evidence for a robust association between muscle phospholipid PUFA content and MRS. In view of the inverse slopes of the relations between MRS and n-6 and n-3 PUFAs (Fig. 1) it is clear that the positive relation of MRS to total PUFAs can be attributed to the relative abundance of n-6 PUFAs alone (which made up ∼75% of all PUFAs). Notably, this relation was detectable even though MRS, which is typically determined by anecdotic observations, is a notoriously error-prone variable [8]. Also, it is remarkable that the strength of this relation to n-6 PUFA content (or to the n-6–n-3 difference) was almost as large as the well known dependency of speed on body weight (which levels off among very large mammals) (e.g., [2], [8]).

Note that we incorporated body weight in our statistical analysis not only to adjust for its effect on MRS, but also because certain fatty acid classes in skeletal muscle show strong allometric relations to body weight. While the proportion of total unsaturates and n-6 PUFA content is quite independent of body mass, n-3 PUFA and DHA content are known to decrease markedly as body size increases [9]. This was also the case in our data set. Therefore, we used a multiple regression approach which tests for the independent effect of each fatty acid class, controlling for correlations between fatty acid content and body weight. In other words, our finding of a significant relation between n-6 PUFAs, or the n-6–n-3 difference, and MRS was not caused by a mere covariation of phospholipid profiles with body size.

We found no direct evidence for positive effects of membrane fatty acids other than n-6 PUFAs, particularly linoleic acid, on MRS. However, the strong negative relation between PUFA and MUFA content (Fig. 2) suggests that species in which dietary uptake of PUFAs is limited compensate for their lack primarily by synthesis and incorporation of MUFAs, rather than SFAs, into muscle phospholipids. An increase of MUFA *de novo* synthesis (particularly of Oleic acid C18:1n-9) when PUFAs are insufficiently present in the diet, has been observed before, and apparently results from the systems involved in regulating membrane acyl composition [10]. Given the positive apparent effect of n-6 PUFAs on MRS, their replacement by MUFAs in muscle membranes must lead to the observed lower running speed in species with high MUFA content. The underlying reason for lacks of PUFAs being filled up by MUFAs may well be however, that phospholipid MUFAs, if their impact could be determined independently from part-whole correlations, actually may turn out to impair MRS less than SFAs.

How could the abundance of n-6 PUFAs, relative to n-3 PUFAs and to MUFAs, in membrane bilayers affect muscle function and hence MRS in mammals? The membranes involved in muscle-cell contraction are the sarcolemma, the SR, and mitochondrial membranes. Arguably, however, mitochondrial respiration and ATP synthesis is not limiting for MRS, because burst muscular activity, as it typically occurs during brief periods of fast running, is governed by anaerobic metabolism [11], [12]. Also, it is clear (for instance, from experimentally induced tetanic contractions) that the rate limiting process which sets the upper limit for contraction frequency is the Ca^2+^ re-uptake into the SR, rather than the generation or propagation of action potentials at the sarcolemma [3]. Therefore, we propose that n-6 PUFAs exert a positive effect on muscle performance and MRS by facilitating Ca^2+^ uptake into the SR by increasing the activity of the sarcoplasmatic Ca^2+^Mg^2+^-ATPase. This conclusion is not only based on the above arguments, but also on experimental findings. Dietary induced changes in SR phospholipid composition in mice indeed altered Ca^2+^Mg^2+^-ATPase activity exactly as it would be predicted from our present observations: An increase in the n-6:n-3 PUFA ratio of SR phospholipids from approximately 1∶1 to 3∶1 caused a more than 5-fold increase in Ca^2+^Mg^2+^-ATPase activity, and a more than 3-fold increase in Ca^2+^ transport into SR vesicles [13]. While these data were obtained from cardiac SR vesicles, the same effect of high proportions of n-6 PUFAs in SR phospholipids significantly increasing Ca^2+^Mg^2+^-ATPase activity was also found in skeletal muscles [14].

Interestingly, these experimental manipulations of SR properties also demonstrated that large changes in Ca^2+^Mg^2+^-ATPase activity occurred in the absence of changes in membrane unsaturation [13], [14]. These observations parallel our present finding of a lack of an influence of fatty acid unsaturation on MRS. This result was somewhat surprising, because well known effects of PUFAs on membrane properties are attributed to their unsaturation. For instance, elevated amounts of the highly unsaturated DHA cause profound increases in the molecular activity of the most ubiquitous ion pump, the Na^+^K^+^-ATPase, as well as significant increases in mitochondrial protein leakage [10], [15], [16]. We suggest that the lack of any relation between phospholipid DHA content (or UI) and MRS in our data, and in the experimental work on mouse SR vesicles [13], is related to the fact that these effects of DHA lead to elevated aerobic metabolism, while MRS, as outlined above, is fuelled by cellular energy stores and anaerobic metabolism. Thus, high amounts of DHA may well have profound effects on the aerobic capacity of, for instance, the high contraction-frequency hummingbird pectoral muscles, which are further augmented by the double packing of mitochondrial inner membranes of this muscle [5]. It is likely that both factors, membrane packing and DHA content, enhance the aerobic capacity of pectoral muscles typically used for sustained flight. These effects of DHA on mitochondrial membranes may, however, be quite unrelated to the positive effects of n-6 PUFAs on burst muscular performance during MRS.

A relatively low mean variation coefficient of muscle phospholipid n-6 PUFA content (10.5%) or of the difference between n-6 and n-3 PUFAs (6.6%) within the mammalian species analysed here, suggests that membrane composition is regulated within species-specific limits. Does this leave sufficient within-species variation to create a selective advantage for individuals with a higher than average muscular n-6 PUFA content? Arguably, it does: Diet can exert a strong influence on skeletal muscle fatty acid composition, in particular on the n-6∶n-3 fatty acid ratio, within a species [17]. Interestingly, dietary manipulation of muscle phospholipid composition resulting in an increased n-6∶n-3 ratio significantly improved exercise performance in rats [17], [18]. Further experimental evidence for a role of n-6 PUFAs in enhancing muscular function comes from dietary fatty acid manipulations in Atlantic salmon which had strong effects on maximum swimming speeds [4]. In line with our observations in mammals, there was a significant increase in swimming speed (R^2^ = 0.978) as the proportion of n-6 PUFAs in the diet and in locomotor muscles increased.

Our results indicate that for each increase in n-6 PUFA content by 1%, MRS increased by approximately 0.2 m s^−1^. If this relation also applies to within-species differences, even a mere 1% increase in muscle phospholipid n-6 PUFAs should translate, in an average mammal, into a 6 meter margin over a 30 second chase at full running speed, which may well determine the survival of flying prey. Our data on European hare, for instance, indicate that actual differences in muscle PUFA content between animals from natural populations can be as large as 30% (29.8%–60.5%). Thus, it seems that the potential effect of muscle phospholipid composition on MRS is strong and constitutes a highly fitness-relevant quality. Arguably, this conclusion should apply to all mammals, except maybe for present-day humans, in which muscle n-6 PUFA content of competing runners might be decisive for the outcome of short-distance races, but will hardly affect their fitness in the evolutionary sense of the word.

## Materials and Methods

### Tissue collection

**Figure 3:**
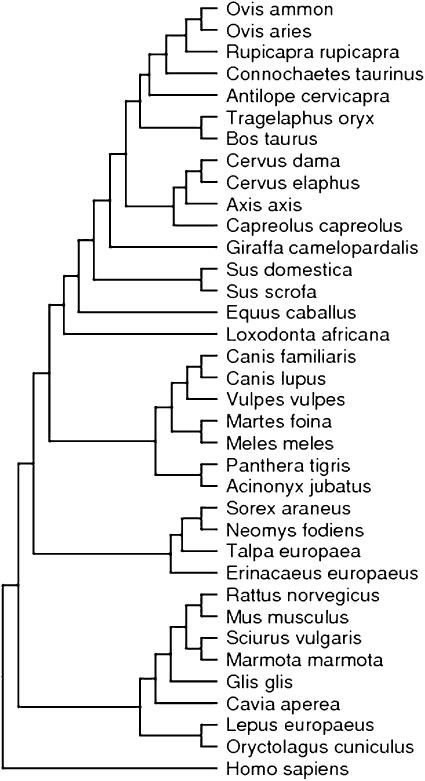
Phylogeny of the mammalian species investigated. Branch lengths are arbitrary.

We compared muscle phospholipid fatty acid content of 36 mammalian species, treating two domestic forms (*Sus domestica* and *Canis familiaris*) like distinct species (Fig. 3). We focused on phospholipids, because they represent the major constituents of cell membrane bilayers. In 9 species, animals were culled during hunting. For 8 species samples were obtained from zoo animals that were killed for population management reasons, or died subsequent to trauma. In 6 species we collected tissues from fresh road kills and in another 4 species, samples were taken from animals from laboratory breeding colonies. We only used fresh tissues without discolorations or gunshot wounds (hunting samples) and road kills that were still warm as outlined in more detail elsewhere [7]. Tissue samples were obtained from a hind leg muscle (*M. vastus*) and stored at −18°C prior to analysis. In small mammals, when we were unable to gain this amount of muscle material from the *M. vastus* alone, we used muscle tissues from the entire hind legs. We felt justified to pool tissues in these cases because muscle (and fibre-type) specific differences in total phospholipid fatty acid profiles are very small compared to the range of between species differences investigated here [7], [19], or not detectable at all [20], [21]. All animals investigated were healthy individuals of both sexes. To avoid any effect of maturational changes in phospholipid composition, we excluded juvenile specimens from our analysis, and only used adult individuals. We tested for, but found no effect of gender on any individual fatty acid or any of the fatty acid classes determined (ANCOVA with adjustment for body weight, P>0.10 in all cases). Details on data and sources are given in Protocol S1.

### Lipid extraction and analysis

Lipids were extracted from muscle samples (0.5 g each) and lipid classes were separated on silica gel thin layer chromatography plates. All solvents contained butylhydroxytoluol in order to avoid oxidative modification of PUFAs. Phospholipids were extracted into hexane, extracts were transesterified under nitrogen, and analysed by GLC (Perkin Elmer Autosystem XL with autosampler and FID; Norwalk, USA) using a capillary column (HP INNOWax, 30 m×0.25 mm; Hewlett Packard, USA). Fatty acid methyl esters were identified by comparing retention times with those of standards (Sigma-Aldrich, St. Louis, USA) and peaks were integrated using the Turbochrom 4.1 Software (Perkin Elmer, Norwalk, USA). Details about chemical analyses and substances used are given elsewhere [7]. For 27 species, we determined the proportions of the following phospholipid acids: C14:0, C15:0, C16:0, C17:0, C18:0, C16:1n-7, C18:1n-9, C18:2n-6, C18:3n-3, C20:4n-6, C20:5n-3, C22:5n-3, and C22:6n-3. Fatty acid percentages were combined into classes of SFA, MUFA, PUFA, UFA according to their degree of unsaturation and, additionally, PUFAs were attributed to the n-3 and n-6 family. For all animals an unsaturation index was computed [22], which represents the average number of double bonds per 100 fatty acid molecules (UI = (%MUFA×1)+(%Dienoic×2)+(%Trienoic×3)+(%Tetraenoic×4)+(%Pentaenoic×5)+(%Hexaenoic×6) ).

Sample sizes within species ranged from 1 to 244 individuals. We felt justified to include single specimens because fatty acid concentrations showed only little to moderate within-species variation (coefficients of variation for all fatty acid (classes) investigated ranged from 2.3% to 36.67%, overall mean: 15.68%). Nonetheless, we computed additional bootstrap statistics (see below) to test for the possible effects of including species for which no replicates were available.

### Literature data

For 9 further species, we used published data on muscle phospholipid composition, in which contents of the same set of fatty acids as determined in our laboratory was given (except for 3 cases in which C14:0, C15:0 or C17:0 had not been determined due to their negligible amount (typically<1%)). We computed mean proportions for each species when more than one source was available. Data on body weight were obtained, if possible, from the actual specimen used for tissue collection (n = 24), or taken from the literature. Also, data on MRS were taken from publications, except for two cases of personal communications of experts on the species in question (see Protocol S1), and one case of using own (TR) unpublished observations on *Glis glis*.

### Statistical analyses

To assess the impact of body weight and phospholipid fatty acid concentration on MRS we used General Additive Mixed Models (GAMM), because this approach allowed us to fit smooth curves (penalized cubic regression splines) to the nonlinear relation of MRS to body weight, and to include possible effects of taxonomic relations. To adjust for phylogenetically caused correlations (e.g., within-clade morphological similarities that may affect MRS), we initially entered order, family, and genus of each species as nested random effects (which corresponds to the nested ANOVA approach for simple linear models). Initial results indicated that inclusion of the factors order and genus decreased the adjusted coefficients of determination and led to over-determined models. Therefore, we included only the taxonomic level family in our final models. Unlike body weight, partial effects of the proportions of fatty acid classes on MRS were best described by simple linear terms (Fig. 1) and were entered as such. GAMMs were computed using the library mgcv [23] within the statistical package R [24]. Because the distribution of MRS was skewed to the right we log transformed MRS. Note that the GAMM models used are an extension of multiple linear regression. Hence, they assess the independent contributions of each independent variable to the prediction of the dependent variable. Thus, using this procedure we statistically eliminated possibly confounding effects of body mass on both MLSP and phospholipid fatty acid composition. We used multiple regression, rather than comparing residuals from individual regressions against body weight because it avoids bias in the estimated coefficients, and computes P-values based on the correct degrees of freedom (with all variables entered into a single model) [25].

To test for the influence of including data with very different levels of uncertainty for each species (i.e., replicates of fatty acid composition ranging from n = 1 to n = 244) we employed a bootstrap procedure. Details on this procedure and its results are given in Protocol S2. To validate our main results on the relation between phospholipid fatty acids and MRS, using more detailed information on the taxonomic relation between the species investigated [26], [27], we computed phylogenetically corrected regressions as outlined in Protocol S3.

## Supporting Information

Protocol S1 Original data and sources.
(0.10 MB DOC)

Protocol S2 Bootstrap analysis of regression models.
(0.02 MB DOC)

Protocol S3 Phylogenetically corrected regressions.
(0.03 MB DOC)
