# Supplementary material for: Running Speed in Mammals Increases with Muscle n-6 Polyunsaturated Fatty Acid Content
Source: PLoS One. 2006 Dec 20;1(1):e65. doi: 10.1371/journal.pone.0000065 (PMC1762323; doi:10.1371/journal.pone.0000065)
Supplement: Protocol S1 — Original data and sources. (0.10 MB DOC) [file pone.0000065.s001.doc]

**Protocol S1.**

Data on muscle phospholipid profiles and body weights were compiled from own measurements (27 species) and published data (9 species). Data on MRS were taken from the literature. In two cases (*Bos tauraus* and *Ovis aries*) MRS was estimated from allometric equations [4]. Details and sources are given in table S1.

**Table S1**. Original data and sources.

|  | **Family** | **Body weight (kg)** | **MRS ^a^ (km h^-1^)** | **SFA (%)^b^** | **MUFA (%)^c^** | **PUFA (%)^d^** | **n-6 (%)^e^** | **n-3  (%)^f^** | **N ^g^** |
| --- | --- | --- | --- | --- | --- | --- | --- | --- | --- |
| **Artiodactyla** |  |  |  |  |  |  |  |  |  |
| *Antilope cervicapra* | Bovidae | 37^1^ | 105^1^ | 24.5* | 7.6 | 67.9 | 56.7 | 11.2 | 2 |
| *Axis axis* | Cervidae | 100* | 45^2^ | 25.9* | 5.3 | 68.8 | 57.1 | 11.8 | 2 |
| *Bos taurus* | Bovidae | 369^3^ | 39^4^ | 29.7^3,5^ | 34.9 | 34.5 | 25.2 | 8.9 | 70 |
| *Capreolus capreolus* | Cervidae | 20* | 60^1^ | 27.1* | 7.6 | 65.4 | 50.3 | 15 | 5 |
| *Cervus dama* | Cervidae | 80* | 65^1^ | 28.1* | 9.5 | 62.4 | 50.6 | 11.8 | 2 |
| *Cervus elaphus* | Cervidae | 150* | 72^1^ | 24.6* | 8.5 | 63.8 | 49.2 | 14.7 | 7 |
| *Connochaetes taurinus* | Bovidae | 165* | 80^1^ | 22.7* | 9.5 | 67.8 | 61.9 | 5.9 | 1 |
| *Giraffa camelopardalis* | Giraffidae | 700* | 60^1^ | 25.8* | 12.9 | 61.3 | 55.4 | 6.9 | 1 |
| *Ovis ammon* | Bovidae | 16.1* | 60^1^ | 20.9* | 11.3 | 67.8 | 48.2 | 19.6 | 1 |
| *Ovis aries* | Bovidae | 32.9^3^ | 34^4^ | 28.6^3^ | 39.9 | 31.5 | 20.2 | 10.3 | 4 |
| *Rupicapra rupicapra* | Bovidae | 35* | 40^1^ | 24.4* | 12.9 | 62.7 | 41.1 | 21.6 | 3 |
| *Sus domesticus* | Suidae | 150^6^ | 18^7^ | 29.3^6,8^ | 18.2 | 52.8 | 49.7 | 3.9 | 40 |
| *Sus scrofa* | Suidae | 50* | 55^9^ | 27.3* | 8.1 | 64.7 | 57.7 | 6.9 | 1 |
| *Tragelaphus oryx* | Bovidae | 400* | 70^1^ | 27.4* | 11.4 | 61.2 | 45 | 16.2 | 1 |
| **Carnivora** |  |  |  |  |  |  |  |  |  |
| *Acinonyx jubatus* | Felidae | 55^1^ | 110^1^ | 27.3* | 22.9 | 49.8 | 42.5 | 7.3 | 2 |
| *Canis familiaris* | Canidae | 20* | 67^1^ | 31.6* | 10.1 | 58.3 | 54.7 | 3.6 | 7 |
| *Canis lupus* | Canidae | 30* | 64^1^ | 28* | 13.1 | 58.8 | 53.8 | 5.1 | 1 |
| *Martes foina* | Mustelidae | 1.58* | 40^10^ | 27.6* | 10 | 62.4 | 54 | 8.3 | 3 |
| *Meles meles* | Mustelidae | 8.50* | 30^1^ | 27.2* | 5.4 | 67.4 | 59.8 | 7.5 | 1 |
| *Panthera tigris* | Felidae | 107* | 56^1^ | 29.1* | 25.6 | 45.2 | 37.5 | 7.7 | 2 |
| *Vulpes vulpes* | Canidae | 5.10* | 42^7^ | 28.9* | 13.3 | 57.8 | 51.2 | 6.6 | 1 |
| **Insectivora** |  |  |  |  |  |  |  |  |  |
| *Erinacaeus europaeus* | Erinaceidae | 1* | 9^11^ | 26.2* | 13.7 | 60.1 | 37.1 | 22.9 | 1 |
| *Neomys fodiens* | Soricidae | 0.01^12^ | 14^13^ | 31.7^12^ | 13.9 | 53.3 | 16.8 | 37 | 8 |
| *Sorex araneus* | Soricidae | 0.01^12^ | 14^13^ | 33.5^12^ | 11.9 | 54.1 | 12.2 | 41.9 | 8 |
| *Talpa europaea* | Talpidae | 0.07* | 4^1^ | 27.2* | 11.3 | 61.6 | 14.8 | 46.8 | 4 |
| **Lagomorpha** |  |  |  |  |  |  |  |  |  |
| *Lepus europaeus* | Leporidae | 3.62^14^ | 72^1^ | 27.3^14^ | 5.7 | 66.7 | 44.3 | 22.5 | 244 |
| *Oryctolagus cuniculus* | Leporidae | 4.10^3,15,16,*^ | 56^1^ | 33.3^3,15,16, *^ | 15.1 | 51.6 | 38.6 | 12.7 | 4 |
| **Perissodactyla** |  |  |  |  |  |  |  |  |  |
| *Equus caballus* | Equidae | 500* | 70^1^ | 28.3* | 7.2 | 64.5 | 57.3 | 7.2 | 6 |
| **Primates** |  |  |  |  |  |  |  |  |  |
| *Homo sapiens* | Hominidae | 80^17^ | 40^1^ | 35.3^17^ | 11.8 | 53.4 | 44.2 | 6.3 | 14 |
| **Proboscidea** |  |  |  |  |  |  |  |  |  |
| *Loxodonta africana* | Elephantidae | 4000* | 35^1^ | 22.7* | 39.2 | 38.2 | 28.6 | 9.6 | 1 |
| **Rodentia** |  |  |  |  |  |  |  |  |  |
| *Cavia aperea* | Caviinae | 0.19* | 12^18^ | 32.9* | 6.1 | 60.9 | 50.3 | 10.7 | 4 |
| *Glis glis* | Gliridae | 0.09* | 10^19^ | 27.2* | 19.9 | 52.9 | 32.6 | 20.3 | 1 |
| *Marmota marmota* | Sciuridae | 4* | 16^1^ | 30.3* | 8.4 | 59.4 | 39.1 | 20.4 | 5 |
| *Mus musculus* | Muridae | 0.04^3^ | 13^1^ | 33.1^3^ | 15 | 51.9 | 30.5 | 20.9 | 4 |
| *Rattus norvegicus* | Muridae | 0.58^3^ | 10^1^ | 35^3^ | 15.1 | 49.9 | 36.5 | 21.18 | 4 |
| *Sciurus vulgaris* | Sciuridae | 0.32* | 20^1^ | 27.1* | 6.4 | 66.6 | 44.5 | 22.1 | 2 |
| **Mean ± SEM** |  |  |  | **28.28**  **± 0.58** | **13.84**  **± 1.46** | **57.52 ± 1.55** | **43.03**  **± 2.22** | **14.81**  **± 1.71** |  |

* this study; ^a^ maximum running speed; ^b^ saturated fatty acids (References given for SFA apply to all fatty acid groups); ^c^ monounsaturated fatty acids;
^d^ polyunsaturated fatty acids; ^e^ n-6 polyunsaturated fatty acids; ^f^ n-3 polyunsaturated fatty acids;
^g^ number of specimens used for fatty acid analysis.

1. Garland T Jr, (1983) The relation between maximal running speed and body mass in terrestrial mammals. J Zool London 199: 157-170.
2. Gambaryan PP (1974) How mammals run. New York: John Wiley and Sons.
3. Couture P, Hulbert AJ (1995) Membrane fatty acid composition of tissues is related to body mass of mammals. J Membrane Biol 148: 27-39.
4. Iriarte-Diaz J (2002) Differential scaling of locomotor performance in small and large terrestrial mammals. J Exp Biol 205: 2897-2908.
5. Laborde FL, Mandell IB, Tosh JJ, Wilton JW, Buchanan-Smith JG (2001) Breed effects on growth performance, carcass characteristics, fatty acid composition and palatability attributes in finishing steers. J Anim Sci 79: 355-365.
6. Muriel E, Ruiz J, Ventanas J, Antequera T (2002) Free-range rearing increases (n-3) PUFAsof neutral and polar lipids in swine muscles. Food Chem 78: 219- 225.
7. Spector WS (1956) Handbook Biological Data. Philadelphia: Saunders.
8. vanLaack RL, Spencer E (1999) Influence of swine genotype on fatty acid composition of phospholipids in longissimus muscle. J Anim Sci 77: 1742-1745.
9. Personal communication: Martys M.
10. Personal communication: Krüger HH.
11. Reeve NJ (1994) Hedgehogs. London: Poyser.
12. Käkelä R, Hyvärinen H (1995) Fatty acids in the triglycerides and phospholipids of the common shrew (*Sorex araneus*) and the water shrew (*Neomys fodiens*). Comp Biochem Physiol B 112: 71-81.
13. Punzo F, Chavez S (2003) Effect of aging on spatial learning and running speed in the shrew (*Cryptotis parva*). J Mammal 84: 1112-1120.
14. Valencak TG, Arnold W, Tataruch F, Ruf T (2003) High content of polyunsaturated fatty acids in muscle phospholipids of a fast runner, the European brown hare (*Lepus* *europaeus*). J Comp Physiol B 173: 695-702.
15. Lopez- Bote CJ, Rey AI, Sanz M, Gray JI, Buckley DJ (1997) Dietary vegetable oils and alpha-tocopherol reduce lipid oxidation in rabbit muscle. J Nutr 127: 1176-1182.
16. Lopez- Bote CJ, Sanz M, Rey A, Castano A, Thos J (1998) Lower lipid oxidation in the muscle of rabbits fed diets containing oats. Anim Feed Sci Technol 70: 1-9.
17. Andersson A, Sjodin A, Hedman A, Olsson R (2000) Fatty acid profile of skeletal muscle phospholipids in trained and untrained young man. Am J Physiol 279: 744-751.
18. Trillmich F, Bieneck M, Geissler E, Bischof H (2003) Ontogeny of running in the wild guinea pig (*Cavia aperea*). J Mamm Biol 68: 214-223.
19. Unpublished data: TR.
