# Supplementary material for: Running Speed in Mammals Increases with Muscle n-6 Polyunsaturated Fatty Acid Content
Source: PLoS One. 2006 Dec 20;1(1):e65. doi: 10.1371/journal.pone.0000065 (PMC1762323; doi:10.1371/journal.pone.0000065)
Supplement: Protocol S2 — Bootstrap analysis of regression models. (0.02 MB DOC) [file pone.0000065.s002.doc]

# Protocol S2

To test for the influence of including data with very different levels of uncertainty for each species (i.e., replicates of fatty acid composition ranging from n=1 to n=244) we employed a bootstrap test with 10 000 runs per predictor variable investigated. For each run, we randomly picked data from only one individual per species (sampled with replacement) and recomputed GAMM regression models as outlined in Material and Methods. Note that this procedure uses both random omission of species during each run and incorporation of all information available on within-species variation for each trait to assess possible bias, particularly that caused by including species represented by single specimens. We provide 95% confidence intervals of the coefficient estimates from this bootstrap procedure for comparison with conventional statistics based on means from each species.

**Table S2.** Results of a bootstrap analysis to validate the relation between muscle phospholipids and MRS.

| **Predictor** | **lower bound** | **mean slope^1^** | **upper bound** |
| --- | --- | --- | --- |
| SFAs | -0.0527 | -0.0245 | 0.0009 |
| MUFAs | **-0.0256^2^** | **-0.0113** | **-0.0016** |
| PUFAs | **+0.0020** | **+0.0096** | **+0.0202** |
| n-6 PUFAs | **+0.0036** | **+0.0109** | **+0.0207** |
| n-3 PUFAs | -0.0265 | -0.0084 | +0.0121 |
| UI | -0.0032 | +0.0018 | +0.0056 |

^1^ Estimated slopes and 95% confidence intervals of repeated GAMM regressions using random picks of individual specimen from each species.

^2^Values in bold face indicate significant (P<0.05) effects.
