# Supplementary material for: Running Speed in Mammals Increases with Muscle n-6 Polyunsaturated Fatty Acid Content
Source: PLoS One. 2006 Dec 20;1(1):e65. doi: 10.1371/journal.pone.0000065 (PMC1762323; doi:10.1371/journal.pone.0000065)
Supplement: Protocol S3 — Phylogenetically corrected regressions. (0.03 MB DOC) [file pone.0000065.s003.doc]

# Protocol S3

To validate results on the relation between phospholipid fatty acids and MRS, we computed phylogenetic generalized least square (GLS) models [1]. This approach is functionally identical to independent contrasts regressions [1]. These models allow the inclusion of detailed information on the taxonomic relations between the species investigated, but have the restriction of assuming linear relations between response and all predictor variables. The distance matrix for these models was constructed from the phylogenetic relationships between the 36 mammalian species investigated shown in Fig. 3. The topology in this dendrogram was constructed combining information on evolutionary relationships given in two recent sources [2,3]. Due to a large number of polytomies, we used arbitrary branch lengths [4] to generate the distance matrix. It has been demonstrated that this use of arbitrary branch lengths has little effect on hypothesis testing in phylogenetic regressions [5]. Multiple linear GLS models with log_10_ transformed values of MRS as the dependent variable, and log_10_ transformed body weights as well as the content of muscle phospholipid fatty acid classes (Protocol S1) as predictor variables (tested separately due to multicollinearities between the classes) were computed with the R-library PHYLOGR [6].

Results from GLS modeling indicated that due to a high degree of similarity within clades, body weight had no independent effect on MRS when adjusted for phylogenetic distances between species. Partial regression coefficients for log_10_ body weight from 6 models (testing for the effects of different fatty acid classes and UI; Table S3) ranged from -0.049 to +0.363 and were not significantly different from 0 (0.372 < P < 0.890). Partial regression coefficients from phylogenetic GLS models for the effects of PUFAs, MUFAs, and n-6 PUFAs, however, were statistically significant (Table S2) and showed the same direction of effects, and similar effect sizes as indicated by our GAMM approach of data analysis.

1. Garland T, Ives AR (2000) Using the past to predict the present: confidence intervals for regression equations in phylogenetic comparative methods. Am Nat 155: 346-364
2. Arnason U, Adegoke JA, Bodin K, Born EW, EsaY et al. (2002) A Mammalian mitogenomic relationships and the root of the eutherian tree. Proc Natl Acad Sci USA 99: 8151-8156.
3. Murphy WJ, Eizirik E, Johnson WE, Zhang YP, Ryder OA et al. (2001). Molecular phylogenetics and the origins of placental mammals. Nature 409: 614-618.
4. Pagel MD (1992) A method for the analysis of comparative data. J Theor Biol 156: 43-442.
5. Garland T, Diaz-Uriarte R (1999) Polytomies and phylogenetically independent contrasts: examination of the bounded degrees of freedom approach. Syst Biol 48: 547-558.
6. Díaz-Uriarte R, Garland T. PHYLOGR: Functions for phylogenetically based statistical analyses. R package version 1.0.4. <http://bioinfo.cnio.es/~rdiaz>

Table S3. Phylogenetic GLS regression results.

| **Predictor^1^** | **Slope** | **SE** | **t** | **P** |
| --- | --- | --- | --- | --- |
| SFAs | -0.0191 | 0.0114 | -1.661 | 0.1060 |
| MUFAs | -0.0101 | 0.0035 | -2.854 | 0.0074 ** |
| PUFAs | +0.0083 | 0.0029 | +2.837 | 0.0077 ** |
| n-6 PUFAs | +0.0098 | 0.0032 | +2.975 | 0.0054 ** |
| n-3 PUFAs | +0.0006 | 0.0073 | +0.089 | 0.9300 |
| UI | +0.0017 | 0.0010 | +1.678 | 0.1028 |

^1^ Due to strong multicollinearities between the fatty acid components separate models were computed with each fatty acid class (or UI) entered as a predictor variable (in addition to body weight) at a time.
